# Sexual Size Dimorphism and Body Condition in the Australasian Gannet

**DOI:** 10.1371/journal.pone.0142653

**Published:** 2015-12-04

**Authors:** Lauren P. Angel, Melanie R. Wells, Marlenne A. Rodríguez-Malagón, Emma Tew, John R. Speakman, John P. Y. Arnould

**Affiliations:** 1 School of Life and Environmental Sciences, Deakin University, Burwood, Victoria, Australia; 2 Institute of Environmental and Biological Sciences, University of Aberdeen, Aberdeen, United Kingdom; Università degli Studi di Milano-Bicocca, ITALY

## Abstract

Sexual size dimorphism is widespread throughout seabird taxa and several drivers leading to its evolution have been hypothesised. While the Australasian Gannet (*Morus serrator*) has previously been considered nominally monomorphic, recent studies have documented sexual segregation in diet and foraging areas, traits often associated with size dimorphism. The present study investigated the sex differences in body mass and structural size of this species at two colonies (Pope’s Eye, PE; Point Danger, PD) in northern Bass Strait, south-eastern Australia. Females were found to be 3.1% and 7.3% heavier (2.74 ± 0.03, *n* = 92; 2.67 ± 0.03 kg, *n* = 43) than males (2.66 ± 0.03, *n* = 92; 2.48 ± 0.03 kg, *n* = 43) at PE and PD, respectively. Females were also larger in wing ulna length (0.8% both colonies) but smaller in bill depth (PE: 2.2%; PD: 1.7%) than males. Despite this dimorphism, a discriminant function provided only mild accuracy in determining sex. A similar degree of dimorphism was also found within breeding pairs, however assortative mating was not apparent at either colony (*R* ^*2*^ < 0.04). Using hydrogen isotope dilution, a body condition index was developed from morphometrics to estimate total body fat (TBF) stores, where TBF(%) = 24.43+1.94*(*body mass*/*wing ulna length*) – 0.58*tarsus length (*r* ^2^ = 0.84, *n* = 15). This index was used to estimate body composition in all sampled individuals. There was no significant difference in TBF(%) between the sexes for any stage of breeding or in any year of the study at either colony suggesting that, despite a greater body mass, females were not in a better condition than males. While the driving mechanism for sexual dimorphism in this species is currently unknown, studies of other Sulids indicate segregation in foraging behaviour, habitat and diet may be a contributing factor.

## Introduction

Dimorphism has evolved in response to selection pressures favouring particular phenotypes. A widespread occurrence across animal taxa, sexual size dimorphism is the morphological difference between males and females of the same species [1]. While some debate surrounds the evolutionary process, three principle hypotheses have been proposed to explain the cause of sexual size dimorphism. The *division of labour* hypothesis relates to males and females within a breeding pair investing their effort into different roles. The *sexual selection* hypothesis focuses on male-male competition for mating opportunities and territorial defence, ultimately favouring larger body size in males [2]. The *food competition* hypothesis relates to ecological causation, whereby differences in body size reduce competition for resources through segregation of prey and habitat use [1]. Many studies have proposed sexual selection as a primary cause, while niche segregation is a consequence and maintainer, of dimorphism [3].

In species where differences in dimorphism between the sexes is not immediately apparent, many studies determine sex based on morphometrics using discriminant function analyses [4–6]. These functions incorporate body mass and other structural measurements to accurately predict sex from calibrations of known sex individuals [7]. While these functions can differ between species and populations [8], they also can be reliable, inexpensive and non-invasive.

Variation in body mass between the sexes may reflect differences in structural size and/or differences in body composition due to relative contribution of fat and lean mass [9]. Body condition can represent energy reserves and, hence, is an indication of an individual’s health and nutritional state [10]. Furthermore, determining body condition is a valuable tool as it can be used to indicate how animals are managing natural environmental variation and stressors [11].

Seabirds are typically referred to as sexually monomorphic [12]. However, there are exceptions, such as the dimorphic Procellariiformes (i.e. petrels and albatross) and penguins [13]. Fairbairn and Shine [12] proposed male-biased dimorphism occurs in species with a large average body mass and in conditions where primary productivity is high. Additionally, selection pressures from competition and flight performance may have caused proportionally larger males [14, 15], with the degree of dimorphism varying between colonies [16–18]. Conversely, some seabird species display reversed sexual size dimorphism with females larger in body mass than males, e.g. frigatebirds and tropic birds [19]. Furthermore, the degree of dimorphism can vary greatly between closely related species [20] as seen in the Family Sulidae, comprised of seven species of boobies (*Sula* spp. and *Papasula* spp.) and three species of gannet (*Morus spp*.) [21].

The boobies display a high degree of reversed sexual dimorphism (10–38% difference), with females being larger in body mass, wing ulna length and culmen length than males of the same species [22–24]. Studies have documented habitat segregation in the foraging behaviour of Sulids [22, 25], indicating food competition as possibly resulting in reversed sexual dimorphism in these species. Differences in body condition between the sexes has also been related to differences in foraging effort due to a smaller body mass in males [26, 27].

In contrast, while subtle differences in plumage have been described [21, 28, 29], gannets have conventionally been considered monomorphic. While Cape gannets (*M*. *capensis*) display distinct dimorphism in the length of the gular stripe, there is strong evidence indicating they are monomorphic in size [30]. In contrast, a recent study has shown northern gannets (*M*. *bassanus*) to be reverse dimorphic with females significantly heavier than males during chick rearing and displaying sexual segregation in foraging behaviour and diet composition [31]. These findings highlight the potential for different selection pressures faced by the sexes, such as their response to climate change.

Populations of Australasian gannet (*M*. *serrator*) in south-eastern Australia, forage in one of the fastest warming regions in the world [32]. Preliminary studies have found sexual segregation in the diet and foraging range of Australasian gannets [33, 34]. As sexual segregation is commonly associated with sexual dimorphism, these differences in Australasian gannets could be explained by sexual dimorphism [35]. Despite the degree of dimorphism being known in other gannet species, it is unknown if the Australasian gannet is size dimorphic. A greater knowledge about the Australasian gannet’s morphology could have implications for adaptive management [36, 37]. Therefore, the aims of the present study were to investigate the degree of sexual dimorphism and body condition of the Australasian Gannet at two breeding colonies.

## Materials and Methods

### Ethics statement

The ethical guidelines of Deakin University Animal Ethics Committee and Animal Welfare Committee were followed during this study. The protocol was approved by Deakin University Animal Ethics Committee (Approvals 86/2010, B20/2013). The project was conducted in accordance with the regulations of the Department of Sustainability and Environment Victoria Wildlife Research (Permit # 10005745, 10006878).

### Study sites and animal handling

**Fig 1:**
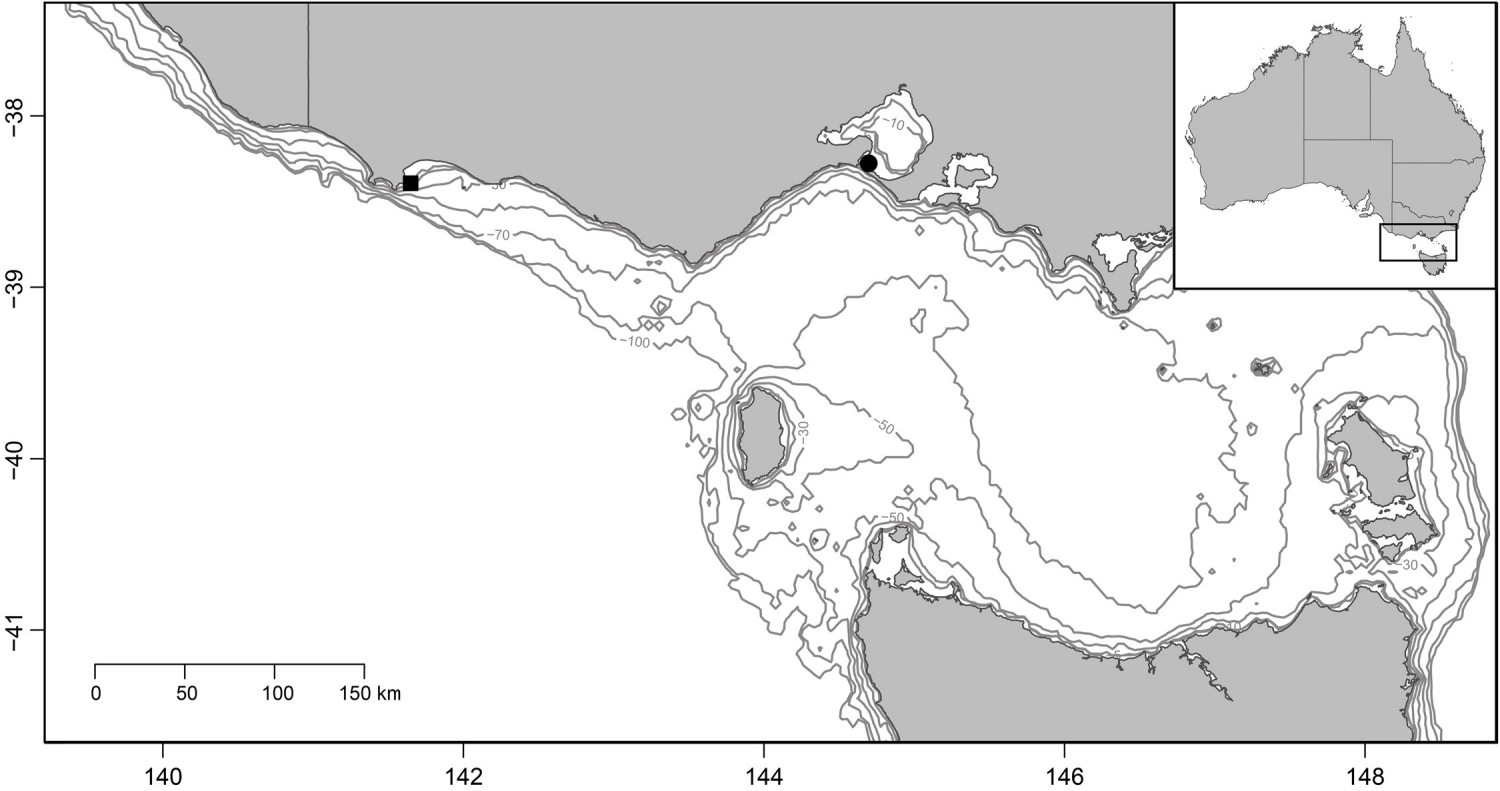
Location of Australasian gannet colonies in the present study, Point Danger (indicated by black square) and Pope’s Eye (indicated by black circle).

The study was conducted over three breeding seasons (2012–2014) at the Pope’s Eye (38°16’42”S 144°41’48”E) and Point Danger (38°23’36”S 141°38’54”E) gannet colonies in northern Bass Strait, south-eastern Australia (Fig 1). A total of 276 Australasian Gannets were weighed and measured at Pope’s Eye (94 pairs) and Point Danger (44 pairs). Sampling was conducted across the breeding season, during incubation and chick rearing. Only pairs were used in the study, with both adults being sampled in the same stage of breeding (sampled either same day or 8 days maximum of each other).

Individuals were captured at the nest (the egg/chick were covered for protection) and weighed using a suspension balance (± 25 g, Salter Australia Pty Ltd, Australia). Incubation and brooding time throughout the breeding season varies from 2 h—5 d (*Angel unpublished data*) and, while the effect of fasting duration can influence body mass, individuals were randomly captured with respect to how long they had been at the nest. Hence, fasting duration is unlikely to have caused a consistent bias in the body mass measurements.

Exposed culmen (bill length), bill depth, and tarsus length were measured using Vernier callipers (± 0.1 mm). Wing chord length could not be measured due to feather deterioration potentially biasing results [5] and, hence, the length of the ulna bone (hereafter, referred to as wing ulna) was measured (± 1 mm) using a slide ruler [38]. Due to logistical constraints, not all measurements were possible on all birds. A blood sample was then collected by venipuncture of the tarsus vein for genetic sexing (DNA Solutions, Australia). Handling time was less than 10 minutes and birds were returned directly onto their nest. Nests were monitored for the remainder of the season.

For each morphometric variable, a size dimorphism index (SDI) was calculated from the mean measurements of males and females, following the methods of Lovich and Gibbons [39], where the extent of dimorphism (percent difference) was calculated as:

$$SDI=\left|-\left(\frac{mean\;male}{mean\;female}\right)+1\right|\times 100$$

### Body composition and condition index

The gross body composition of a sub-sample of individuals was determined in order to develop a body condition index from morphometric measurements. Following measurements of mass and morphometrics, a 0.5 mL background blood sample was collected into a heparinised syringed by venipuncture of the tarsus vein (to determine the background levels of ^2^H) before individuals were administered an intraperitoneal injection of 1.74 ± 0.03 mL ^2^H_2_O (34.1% AP). They were returned to the nest for 3.4 ± 0.1 h as an isotope equilibration period, before another blood sample was collected to determine the size of the total body water pool [40]. Previous studies have found labelled hydrogen to equilibrate with the body water pool within 2 h [41, 42]. Samples were stored (4°C) for several hours before the plasma and red cell fractions were separated by centrifugation and aliquots (50–75 µL) of plasma stored in flame sealed capillary tubes until analysis.

In the laboratory, capillaries that contained the plasma samples were vacuum distilled, and the water from the resulting distillate was used to produce H_2_. The isotope ratio ^2^H:^1^H was analysed using gas source isotope ratio mass spectrometry (Isoprime IRMS and Isochrom mG; Micromass, Manchester, United Kingdom). The isotope dilution space was calculated using the plateau method [43]. Previous studies have found that hydrogen isotope dilution overestimates the total body water (TBW) pool [44], as such, the isotope dilution space was corrected by 3% to take this into account. Using previously published relationships of gross chemical body composition [45], the following calculations were used to determine lean body mass (LBM) and total body fat (TBF) from body mass (BM) and TBW:

LMB(kg)=TBW(kg)/0.73

TBF(kg)=BM(kg)−LBM(kg)

TBF(%)=TBF(kg)/BM(kg)×100

The initial calculation for LBM takes into account the hydration constant typical for birds (0.73) described by Mata, Caloin [11]. A general linear model (GLM) was then constructed using *a priori* knowledge of actual body condition to develop a condition index for all individuals where linear measurements were complete, as well as body mass divided by each measurement. Model selection was based on second-order Akaike information criteria and model averaging. The resulting best fit model describing TBF(%) content from mass and morphometric measurements was then used to assess sex, stage and year effects on body condition.

The assumptions of independence and normal distribution were tested with a Chi-Square test and Shapiro–Wilk’s test, respectively. Differences between the sexes were analysed with a three-way ANOVA with sex, stage and year as interaction terms. Differences within breeding pairs were analysed with a paired t-test. Analysis was carried out using the R statistical environment 3.1.3 and results are reported as Mean ± SE.

## Results

### Body mass and morphometrics

Body mass did not differ between stages (*F*_1,249_ = 0.76, *P* = 0.38) or years (*F*_1,249_ = 2.34, *P* = 0.10) but was found to differ significantly between the two colonies (ANOVA, *F*_1,249_ = 14.38, *P* < 0.001). Additionally, body mass significantly differed between the sexes, with females being consistently heavier across stages and years than males, at both Pope’s Eye (2.74 ± 0.03 *vs* 2.66 ± 0.03, 3.1% larger, *F*_*1*,*183*_ = 4.81, *P* = 0.03) and Point Danger (2.67 ± 0.03 kg *vs* 2.48 ± 0.03 kg, 7.3% larger, *F*_*1*,*84*_ = 24.75, *P* < 0.001; Table 1). While tarsus length (Pope’s Eye: *F*_1,144_ = 0.22, *P* = 0.64; Point Danger: *F*_1,49_ = 0.06, *P* = 0.81) and bill length (Pope’s Eye: *F*_1,146_ = 0.47, *P* = 0.50; Point Danger: *F*_1,72_ = 0.01, *P* = 0.91) were not significantly different between the sexes, females were found to have smaller bill depth (2.2% smaller, *F*_*1*_,_*145*_ = 10.42, *P* = 0.002) but larger wing ulna (0.8% larger, *F*_*1*_,_*147*_ = 45.01, *P* = 0.03) at Pope’s Eye. While the degrees in variation between the sexes in bill depth and wing ulna were of similar magnitude and direction at Point Danger (1.7% and 0.8%, respectively), the differences were not significant (*F*_*1*_,_*72*_ = 2.25, *P* = 0.14; *F*_*1*_,_*72*_ = 1.89, *P* = 0.17). However, this could be due to the smaller sample size at this colony as a power analysis revealed that, for a 1-β = 0.8, sample sizes of 65 and 88 pairs would be sufficient to find a significant difference in bill depth and wing ulna length, respectively, at Point Danger.

**Table 1:** Mean body mass (kg) and morphometrics (mm) ± SE for male and female Australasian Gannets measured at Pope’s Eye and Point Danger colonies, south-eastern Australia. Sexual dimorphism index (SDI) indicates percentage of difference between the sexes.

		Male	*n*	Female	*n*	SDI
Pope’s Eye	Body mass*	2.66±0.03	93	2.74±0.03	92	3.1
	Bill depth*	33.3±0.2	85	32.6±0.2	71	-2.2
	Bill length	92.2±0.3	85	91.7±0.3	73	-0.6
	Wing ulna*	202.6±0.4	85	204.2±0.6	74	0.8
	Tarsus	67.9±0.3	82	67.8±0.3	74	-0.7
Point Danger	Body mass*	2.48±0.03	43	2.67±0.03	43	7.3
	Bill depth	32.8±0.2	41	32.3±0.3	43	-1.7
	Bill length	92.3±0.4	41	90.86±1.28	43	-1.5
	Wing ulna	202.8±0.7	41	204.3±0.9	43	0.8
	Tarsus	69.0±0.5	29	68.8±0.5	31	-0.3

*Significant difference between sexes (P<0.05)

To assess whether the degree of sexual size dimorphism was sufficient to accurately determine sex from morphometric measurements, a discriminant function was developed. A discriminant score (DS) was calculated for each study site as the morphometric variables and the degree of sexual dimorphism can differ between colonies in seabirds [8, 16]. Significant discriminant functions were developed for Pope’s Eye (Wilks’ λ = 0.88, *F*_6,151_ = 13.31, *P* < 0.01):

DS=(−1.83×bodymass)+(0.57×billdepth)+(−0.13×wingulna)+(0.15×tarsus)

and Point Danger (Wilks’ λ = 0.70, *F*_6,59_ = 19.52, *P* < 0.01):

DS=(−4.71×bodymass)+(0.24×billdepth)+(−0.07×billlength)+(−0.05×wingulna)+(0.08×tarsus)

**Fig 2:**
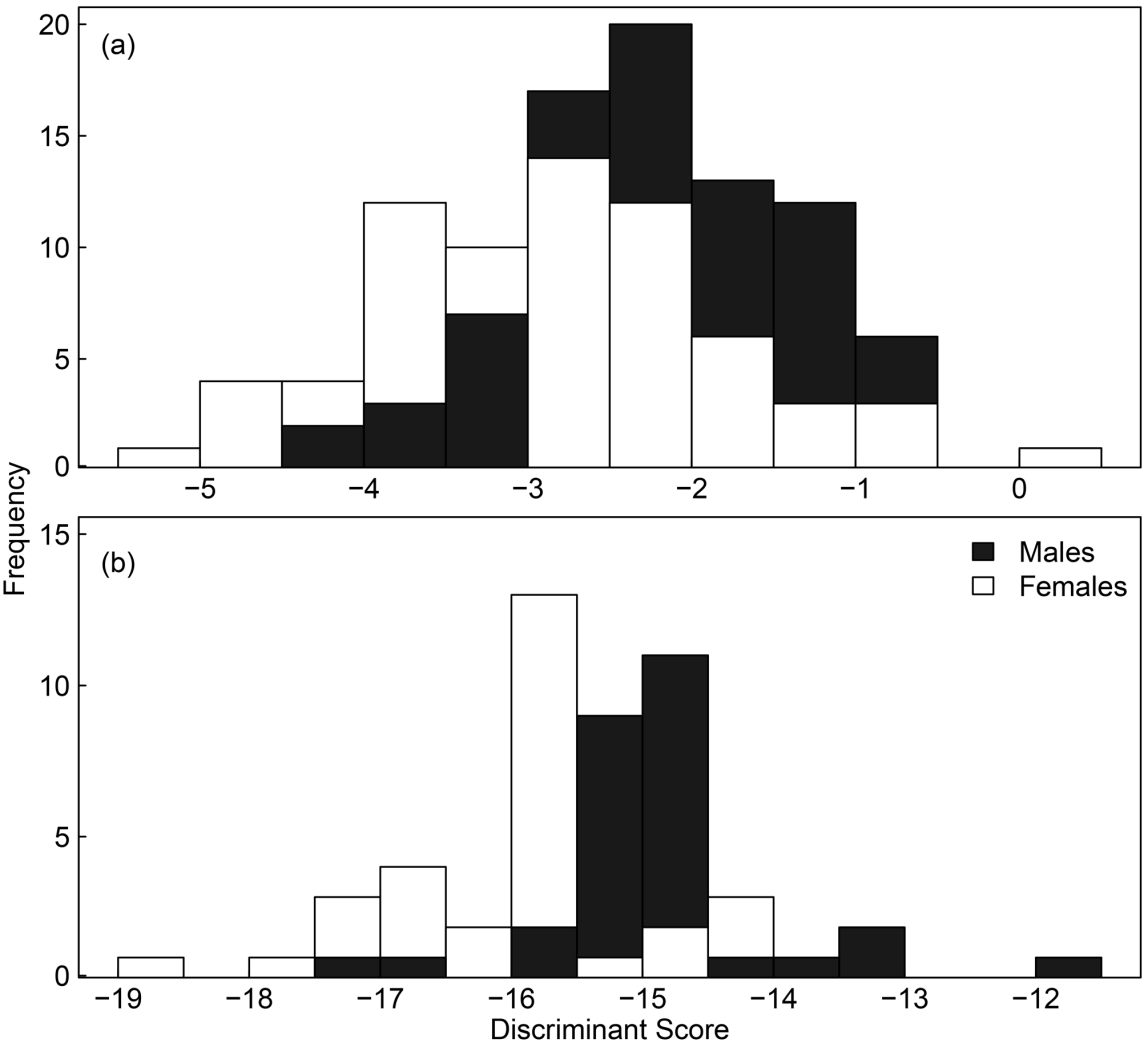
Distribution of discriminant scores for male and female Australasian gannets at two breeding colonies. Scores given for (a) Pope’s Eye and (b) Point Danger colonies, where males are in black, and females are in white. Scores are based on the discriminant functions provided in text.

The predictive accuracy of the discriminant function was 81.4% for Point Danger but 68.2% for Pope’s Eye due to high overlap of scores between sexes (Fig 2).

The degree of sexual dimorphism was also assessed within breeding pairs at both colonies. Individuals within pairs at Pope’s Eye differed significantly in body mass (Paired-samples *t*-test, *t*_*90*_ = 2.30, *P* = 0.02) with females heavier than their partner in 62% of cases. A significant difference in bill depth (*t*_*67*_ = -3.24, P = 0.002) and wing ulna length (*t*_*70*_ = 2.04, P = 0.05; Table 2) were also found. In contrast, individuals in pairs at Point Danger only differed significantly in body mass (*t*_41_ = 5.62, *P* < 0.001), with females heavier than males in 83% of pairs. On average, females were 2.2% heavier than their partner at Pope’s Eye (82 ± 3 g; range: -24.0–33.6 g difference) and 7.3% heavier at Point Danger (203 ± 3 g; range: -12.9–27.5 g difference). The sexual dimorphism index suggests intra-colony and intra-pair dimorphism was similar at Pope’s Eye (SDI values Table 1 and Table 2). At Point Danger, bill depth and bill length was more dimorphic within breeding pairs than within the colony, however assortative mating was not apparent in males or females for these variables (bill depth: *R* ^*2*^ = 0.01, *F*_1,38_ = 0.55, *P* = 0.46; bill length: *R* ^*2*^ < 0.001, *F*_1,38_ = 0.04, *P* = 0.85). Furthermore, there was no evidence for assortative mating in relation to body mass at either colony as no correlation was found between female and male mass within pairs (Pope’s Eye: *R* ^*2*^ = 0.04, *F*_1,89_ = 3.44, *P* = 0.07; Point Danger: *R* ^*2*^ = 0.03, *F*_1,40_ = 1.37, *P* = 0.25). Additionally, no relationship was found in body condition within pairs (Pope’s Eye: *R* ^*2*^ < 0.001, *F*_1,58_ = 0.02, *P* = 0.89; Point Danger: *R* ^*2*^ = 0.03, *F*_1,24_ = 0.61, *P* = 0.44).

**Table 2:** Comparison of mean body mass (kg) and morphometric (mm) differences ± SE within breeding pairs (*n*) of Australasian Gannets from Pope’s Eye and Point Danger colonies.

		Mean difference	*n*	SDI
Pope’s Eye	Mass*	0.08±0.04	91	2.2
	Bill depth*	-0.8±0.2	68	-2.6
	Bill length	-0.6±0.5	70	-0.7
	Wing ulna*	1.6±0.2	71	0.7
	Tarsus	-0.1±0.5	61	-0.4
Point Danger	Mass*	0.20±0.04	42	7.3
	Bill depth	-0.6±0.4	41	-2.1
	Bill length	-1.5±1.4	41	-3.5
	Wing ulna	1.4±0.1	41	0.8
	Tarsus	-0.6±0.7	26	-1.1

*Significant difference within pairs (P<0.05)

### Body condition index

Gross body composition data were obtained from a total of 15 individuals (4 females, 11 males). Total body fat (TBF; %) ranged from 5.6–18.5% (10.5 ± 1.0) and was not significantly different between the sexes (*F*_*1*,*13*_ = 1.58, *P* = 0.23). The top-ranked statistical models explaining TBF from linear morphometrics were determined (Table 3). However, as the combined weight of the models was low (*ω*_*i*_ > 0.9), the coefficients and standard errors were calculated using model averaging. Tarsus length and wing ulna length were selected as the most important variables. While the top two models included both these variables, the most parsimonious model describing the relationship between TBF(%) and morphometric measurements was chosen as:

$$TBF\left(\%\right)=24.43+1.94\times \left(\frac{BM}{WU}\right)-0.58\times T$$

**Table 3:** Top-ranked AIC_c_ models for explaining total body fat(%) from morphometric measurements in adult Australasian gannets (ΔAIC < 4.0). Body mass (kg), wing ulna and tarsus (mm) were selected as important variables by the model.

Model	AICc	ΔAIC	AIC Weight
(body mass/wing ulna) - tarsus	65.0	0.00	0.256
(body mass/tarsus) - wing ulna	65.1	0.10	0.243
body mass/tarsus	66.3	1.30	0.137
body mass - tarsus	67.9	2.88	0.061
(body mass/tarsus) - tarsus	68.1	3.03	0.056

**Fig 3:**
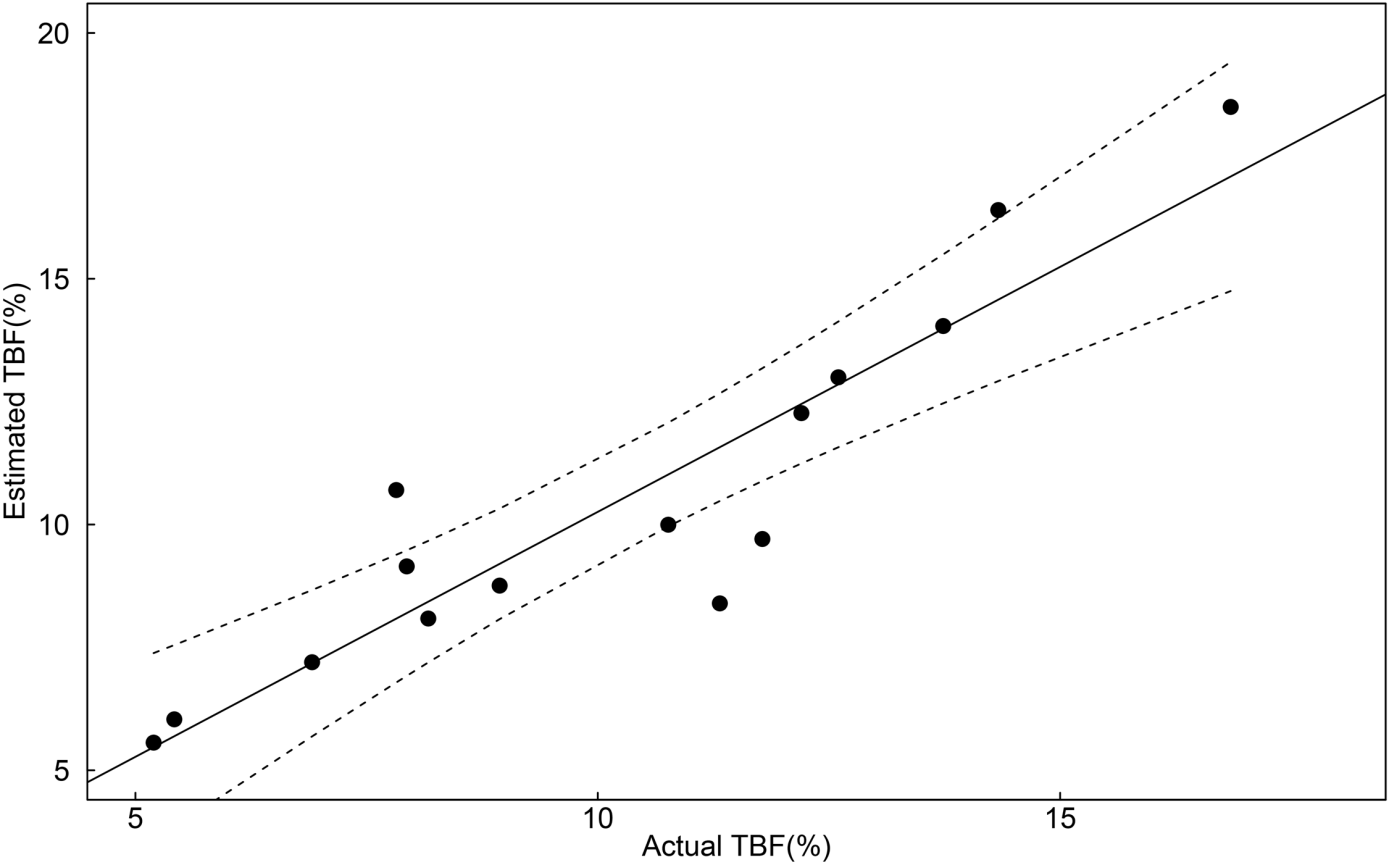
Estimated total body fat(%) reliably predicts actual total body fat(%) in Australasian gannets based on a body condition index. Measurements of body mass (kg), wing ulna and tarsus length (mm) were selected. Plot shows the predicted model (solid line) and the 95% confidence interval (dashed line).

**Fig 4:**
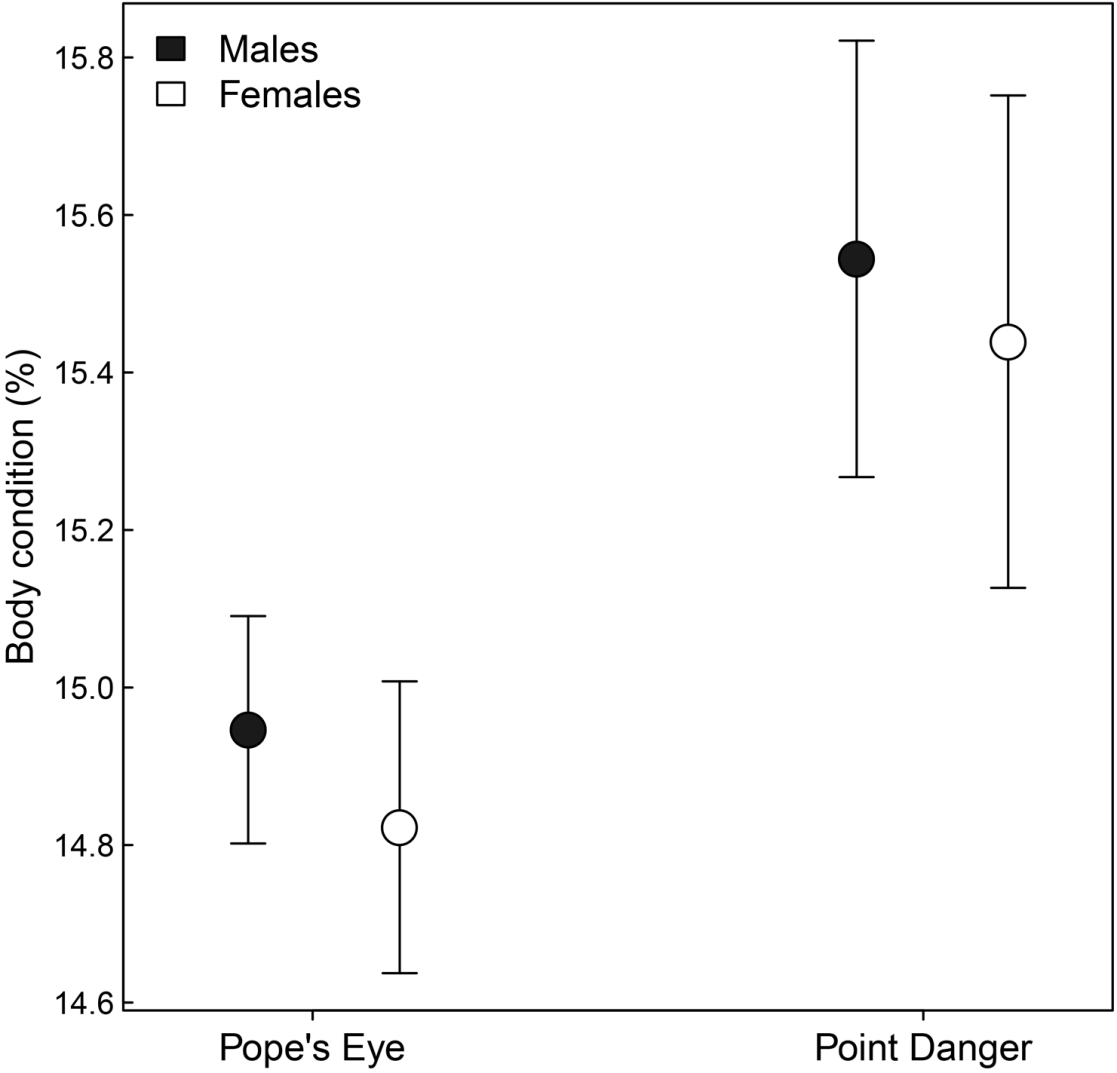
Mean body condition (%) ± SE for males and females from Pope’s Eye and Point Danger gannet colonies. Where males are in black and females are in white.

Where BM is body mass (kg), WU is wing ulna (mm) and T is tarsus (mm). The predicted TBF(%) was highly correlated to the measured TBF (*r* ^*2*^ = 0.84; Fig 3) indicating the model can be used as a valid body condition index (BCI). This BCI was then used to determine the condition of individuals in which the predictor variables were measured from Pope’s Eye (range: 10.1–17.7%; n = 153) and Point Danger (range: 12.8–21.0%; n = 59). At both colonies, BCI did not differ between years (Pope’s Eye: *F*_*2*,*143*_ = 0.13, *P* = 0.88; Point Danger: *F*_*2*,*49*_ = 1.11, *P* = 0.34) or breeding stages (Pope’s Eye: *F*_1,143_ = 0.08, *P* = .078, Point Danger: *F*_1,49_ = 2.14, *P* = 0.15) and, thus, data were combined. The BCI was not significantly different between males (14.6 ± 0.1%) and females (14.5 ± 0.2%) at Pope’s Eye (*F*_*1*,*143*_ = 0.28, *P* = 0.60; Fig 4). Similarly, males (15.2 ± 0.3%) and females (15.1 ± 0.3%) at Point Danger were of a similar BCI (*F*_*1*,*49*_ = 0.06, *P* = 0.80). Additionally, no difference in BCI was found within breeding pairs (Pope’s Eye: *t*_*59*_ = 0.31, *P* = 0.76; Point Danger: *t*_*25*_ = 0.87, *P* = 0.39).

## Discussion

### Body mass and morphometrics

Within the Sulids, boobies display reverse sexual dimorphism with females larger than males by 10–38% (depending on species) [21]; whereas gannets have nominally been considered monomorphic. Recently, however, the northern gannet has been shown to also display reversed dimorphism, with females 7–8% heavier than males [31, 46]. In addition, females of this species travel further from the colony and target different prey items [31, 47] suggesting ecological effects resulting from this dimorphism. As Sulids are a closely related group [48], the presence of reverse sexual dimorphism might, therefore, be expected to some degree in all species. However, there is strong evidence indicating they while the Cape gannet displays distinct dimorphism in the length of the gular stripe, it has been shown to be size monomorphic, with no significant difference in the average body mass of males and females [30]. Hence, the degree of dimorphism across the gannet species, and the influence this has on their behaviour is of interest. The results of the present study, with females at both study colonies being heavier than males consistently across years and breeding stages, confirm the nominally monomorphic Australasian gannet also exhibits reversed sexual dimorphism.

Interestingly, the degree of body mass dimorphism was much greater at Point Danger (7.3%) than at Pope’s Eye (3.1%). The degree of sexual dimorphism can vary geographically within species, for example, in little penguins (*Eudyptula* minor) the dimorphism in bill depth varies considerably between colonies in both Australia and New Zealand [16, 49]. Such differences in the degree of dimorphism may be due to food availability or inter-population growth patterns [39]. Alternatively, sexual selection may be driving the greater divergence in dimorphism [50].

In the present study, females were found to be structurally larger in wing ulna length (0.8% larger at both colonies), indicative of a slightly greater overall body size [38]. This is consistent with findings in brown (*S*. *leucogaster*), red-footed (*S*. *sula*) and Peruvian (*S*. *variegata*) boobies where females have a greater wing cord length (2.8–3.1% larger) than males [51, 52]. Wing size and shape must be optimal to provide enough lift to support body mass in flight and, consequently, variations in wing morphology can alter flight behaviours [53]. Thus, a difference in the wing morphology between sexes, however slight, may have energetic consequences for foraging strategies [54]. As a plunge diving species, take-off from the sea surface is considered the most energetically expensive activity while foraging [55]. With a greater body mass, females may require greater effort to take-off compared to males and a different wing morphology may assist in this.

Consistent with the findings in Australasian gannets in New Zealand [56], males in the present study had a greater bill depth at Pope’s Eye (2.2% larger) and Point Danger (1.7%). The development of larger bills in males may have evolved for nest defence against conspecifics, courtship ritual (bill fencing in Sulids) [21], or to reduce intra-specific competition by enabling them to target prey of a larger size [57]. If the larger bill depth observed in males allows them to target different prey, they may consume a greater proportion of larger prey items/species [58]. Indeed, female blue-footed boobies (*S*. *nebouxii*) possess a larger bill length/depth than males and consume similar prey species but target individuals of a larger size [59]. Prey size and species consumed by Australasian gannets have previously been reported [60], however differences between the sexes are currently unknown.

In a species with no obvious differences in their plumage or body size between sexes, the use of behavioural observations (e.g. copulation, egg-laying by females, or nape-biting by males) have been used to determine the sex of individuals [61]. While effective, this method is usually only applicable at the start of the breeding season. Alternatively, discriminant functions have been widely used in seabird species to determine the sex of individuals in the field from morphometric measurements with an accuracy >90% [4, 6, 62]. However, the applicability of a discriminant function across a species is not always possible and can depend on the degree of variation in morphometrics between colonies [16]. In the present study, a broad range of body sizes were found within the sexes at both colonies. With no clear bimodal distribution of morphometrics by sex, the accuracy of the function was weak at Pope’s Eye (68.2%) and, although it was more accurate at Point Danger (81.4%), it appears DNA analysis is still the most reliable method for determining sex in the Australasian gannet.

In the present study, the degree of dimorphism in Australasian gannets was also investigated within pairs with similar results to that found when comparing intra-colony differences. Females were significantly heavier than their partner at both colonies, with bill depth and wing ulna only significantly different at Pope’s Eye. As body size and structure can evolve due to the need to exploit different niches, dimorphism within pairs can result in a greater exploitation of a diverse array of resources [19]. Previous studies on Sulids have found females forage further from the colony than males, depending on breeding stage [25, 31]. This indicates a niche segregation between the sexes, with males possibly remaining closer to the colony to maintain territory and females foraging further but contributing a greater proportion to the chicks’ diet [63]. Interestingly, no assortative mating was found in the present study, suggesting females are not selecting mates based on body size or condition. However, as gannets are long lived and monogamous [21], size convergence after pairing could occur, with the condition of an individual influencing the traits of their partner over time [64].

### Body condition

Body condition, pertaining to an animals’ fat reserves, can be used as a proxy for individual investment in offspring [65] or reflective of environmental fluctuations in foraging conditions [66, 67]. Body condition is often presented as a body condition index (BCI) based on an individual’s body mass relative to its structural size [68], although the accuracy of the BCI varies between species [69]. Additionally, the linear morphometric measurements used to calculate BCI can strongly affect the relationship. As presented in Schamber, Esler [70], if an unverified measurement is used, despite being verified in another species, false conclusions can be made about body condition. While widely used in seabirds [26, 71, 72], body condition indices are very rarely validated [44, 73].

In Sulids, no validation of body condition as a measure of total body fat exists. Studies relating to body condition report either body mass [74, 75] or an index with body mass regressed against wing length, tarsus or culmen length [26, 76–78]. Furthermore, across studies of the same species these indices have not been consistent and, hence, comparison is not possible. In the present study, a BCI derived from body mass and two structural measurements (wing ulna and tarsus length) was found to be highly correlated to empirical measures of total body fat (%). Unfortunately, the sample size for the isotope dilution study was limited. While sex did not influence the model, it is possible that with a larger sample size this could have been evident. However, the development of the body condition index relates to how body fat content is reflected in morphometric variables and, as such, physiologically it would likely not be impacted by sex. This validated BCI, presents a quick monitoring assessment tool with potential applicability to other gannet and Sulid species.

The TBF(%) of Australasian gannets ranged between 10.1–17.7% at Pope’s Eye and 12.8–21.0% at Point Danger. As no fat content data is available in Sulids these results were compared to other seabirds. Body fat in the black-legged kittiwake (*Rissa tridactyla*) has been reported as 4–13% of the total body mass [73], while Shaffer, Gabrielsen [44] found glaucous gulls (*Larus hyperboreus*) had an average fat content of 3.6 ± 2.6%. The higher body fat of gannets may be reflective of their high calorific diet [60].

In the present study, female gannets were in a similar condition to males, both across the population and within breeding pairs. This may indicate that there is equal effort invested in rearing offspring between the sexes [25]. Lormee, Jouventin [26] found the BCI of red-footed boobies remained stable throughout the breeding season, yet Weimerskirch, Corre [25] found males lost condition faster than females, probably due to the smaller body size of males. Cape gannets, nominally monomorphic in body size, have been reported to lose condition throughout the breeding season in both sexes, although females remain in a better condition than males [79]. As body condition varied in dimorphic and monomorphic species, body size may not be the sole reason for variation in body fat between the sexes. Schultner, Kitaysky [80] suggested birds may choose to maintain lower energy/fat stores than they can physiologically possess in an effort to reduce flight costs, a factor of equal importance to both sexes. Similarly, body fat stores act as buoyancy for diving birds and, can thus reduce the depths and durations to which they can attain [81]. Attaining the necessary depths to predate with minimal effort would be equally beneficial to both sexes and may explain the lack of difference in body condition observed in Australasian gannets.

During the course of the present study, breeding success (% chicks fledged)[82] measured for the entire colony varied greatly, being lower in 2013 (8.6 and 0.0%) than in 2012 (30.1 and 10.0%) and 2014 (24.7 and 48%), at Pope’s Eye and Point Danger, respectively (*Angel unpublished data*). The lower breeding success is suggestive of poor prey conditions in 2013 [83] yet no difference in body condition was found for any stages between years throughout the study. Cape gannets have also been found to maintain a stable body condition across years of differing environmental conditions [77, 79]. This is consistent with gannets, being long-lived, prioritising their own survival (i.e. maintain body condition) when food availability drops below a critical threshold, at the expense of the current breeding attempt [84].

As females are larger in body mass and wing ulna length, yet possess proportionally similar body fat content to males, this suggests females are simply proportionally larger than males. The *sexual selection* hypothesis suggest that in reverse dimorphic species a larger body size in females may be indicative of an individual’s ability to produce larger eggs [85]. However, gannet egg size and mass shows very little variation [86, 87]. The *division of labour* hypothesis has been proposed as a potential factor in dimorphism of booby species [22–24, 59]. However, male and female gannets spend similar time away from the nest foraging [56]. Sexual dimorphism in Australasian gannets may, therefore, be due to *food competition*. As with other Sulids [22, 25, 31, 46, 51], preliminary studies of Australasian gannets have found sexual segregation in foraging range, habitat and diet [34, 35]. Consequently, future studies of foraging behaviour in gannets should assess males and females separately as dimorphism can have ecological consequences.

## Supporting Information

S1 File Morphometric measurements for all individuals used in the present study. (XLSX)
